# Isolated myeloid sarcoma presenting with small bowel obstruction: a case report

**DOI:** 10.1186/s40792-019-0759-6

**Published:** 2020-01-03

**Authors:** Rie Mizumoto, Masanori Tsujie, Tomoko Wakasa, Kotaro Kitani, Hironobu Manabe, Shuichi Fukuda, Kaoru Okada, Shumpei Satoi, Hajime Ishikawa, Toshihiko Kawasaki, Hitoshi Hanamoto, Masao Yukawa, Masatoshi Inoue

**Affiliations:** 1 Department of Surgery, Kindai University Nara Hospital, 1248-1 Otoda, Ikoma, Nara, 630-0293 Japan; 2 Department of Pathology and Laboratory Medicine, Kindai University Nara Hospital, 1248-1 Otoda, Ikoma, Nara, 630-0293 Japan; 3 Department of Gastroenterology and Hepatology, Kindai University Nara Hospital, 1248-1 Otoda, Ikoma, Nara, 630-0293 Japan; 4 Department of Hematology, Kindai University Nara Hospital, 1248-1 Otoda, Ikoma, Nara, 630-0293 Japan

**Keywords:** Myeloid sarcoma, Intestine, Chemotherapy

## Abstract

**Background:**

Myeloid sarcoma (MS) is a solid tumor consisting of myeloid blasts or immature myeloid cells, which are unusual outside the bone marrow.

**Case presentation:**

We present a rare case of isolated myeloid sarcoma of the small bowel in a 54-year-old man who was admitted to our hospital with repeated symptoms of intestinal obstruction. A small bowel series via an ileus tube revealed severe jejunal obstruction. Computed tomography revealed that the obstruction was likely caused by a jejunal tumor. The patient underwent laparoscopy-assisted partial resection of the jejunum with lymphadenectomy. Histopathological examination of the surgical specimen confirmed that MS had been responsible for the obstruction.

**Conclusions:**

Patients with MS require systemic chemotherapy, as do patients with acute myeloid leukemia. Hence, an early, accurate diagnosis is imperative for treating this malignancy. It is also important to list MS in the differential diagnosis of a small bowel tumor, even in nonleukemic patients.

## Background

Myeloid sarcoma (MS), a solid tumor consisting of myeloid blasts or immature myeloid cells outside the bone marrow, is an unusual presentation of acute myeloid leukemia (AML). MS is also known by other names—e.g., chloroma, granulocytic sarcoma—which can create some confusion in understanding this disease. MS can develop anywhere in the body, with the most common sites being the lymph nodes, bone/spine, and skin. Development of MS in the small intestine is reported to account for 10–11% of all MSs occurring in the gastrointestinal tract [1]. Because of its rarity and difficult diagnosis, MS has often been misdiagnosed as other diseases [2]. Here, we report a rare case of isolated primary MS of the small bowel causing intestinal obstruction.

## Case presentation

**Fig. 1:**
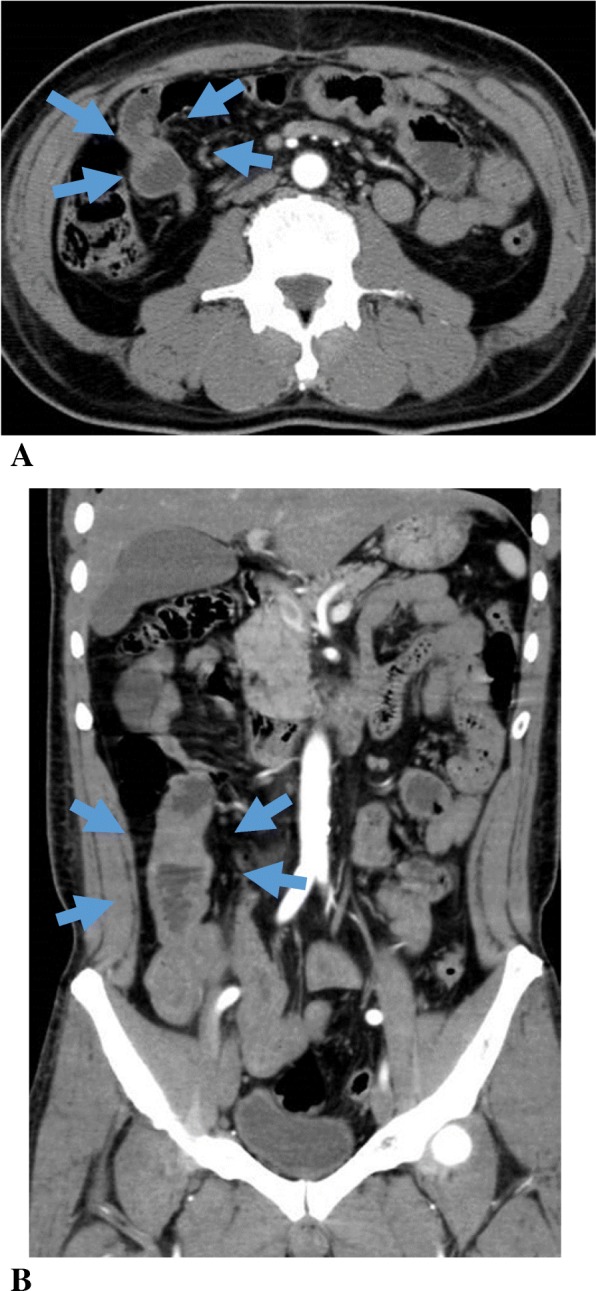
Contrast-enhanced abdominal computed tomography shows thickening of the small bowel wall. **a** Axial view. **b** Coronal view

A 54-year-old man with a history of hypertension and hyperlipidemia was admitted to our hospital complaining of abdominal pain and vomiting. He showed abdominal bloating but no signs of peritoneal irritation. Contrast-enhanced computed tomography (CT) revealed local thickening of the small bowel wall, which was assumed to be due to an inflammatory reaction (Fig. 1). We diagnosed inflammatory disease, administered conservative treatment, which was effective, and did not perform further checkups. After resuming oral intake, he was discharged 10 days after admission. A few days after discharge, however, his abdominal symptoms recurred, and he was readmitted to our hospital.

**Fig. 2:**
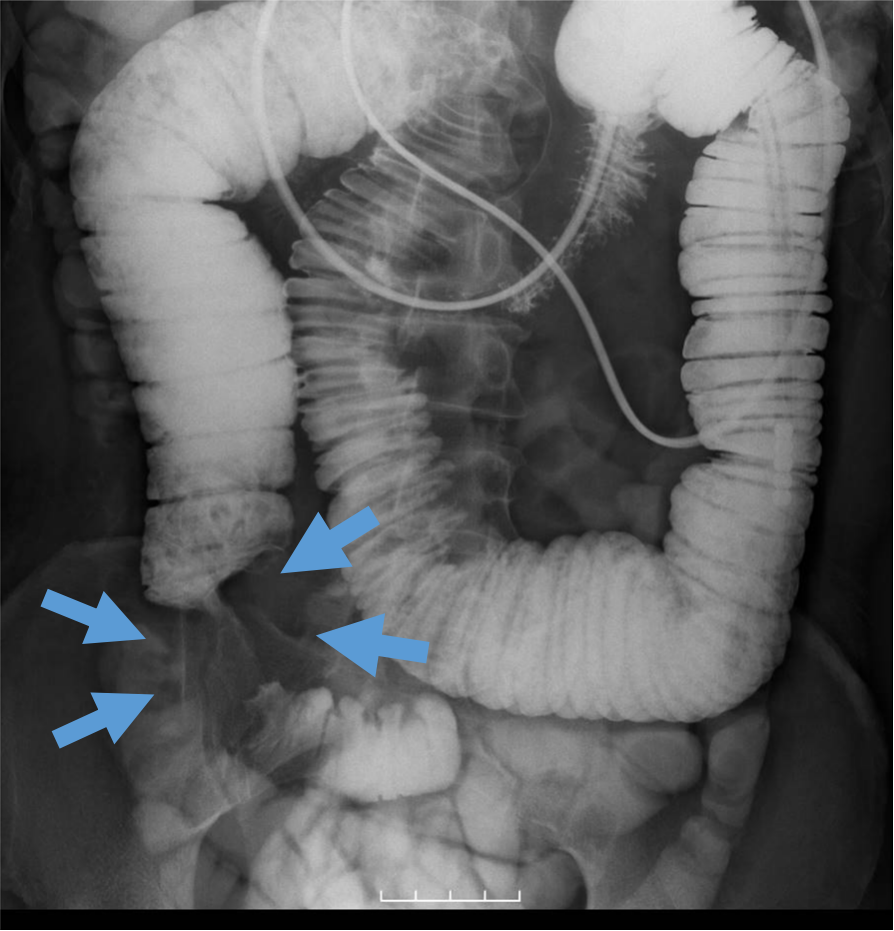
Small bowel series via the ileus tube shows severe obstruction in the jejunum with upstream dilatation

Blood tests showed a mild inflammatory reaction and dehydration. The white blood cell count was 8200/μl, with 62.3% neutrophils, 28.3% lymphocytes, 0.7% eosinophils, 0.5% basophils, and 8.3% monocytes. Other laboratory tests showed the following: hemoglobin 14.5 g/dl, platelets 265,000/μl, CEA 7.5 ng/ml, CA19-9 4.5 U/ml, and sIL-2R 313 U/ml. After insertion of an ileus tube, a small bowel series revealed severe obstruction in the jejunum with upstream dilatation (Fig. 2). CT showed that thickening of the jejunal wall was still causing obstruction. Because of the results of those assessments, we performed a laparoscopic exploratory examination for a more accurate diagnosis. The laparoscopic views revealed a hard mass at the stenotic site, prompting us to perform small bowel resection with mesenteric lymph node dissection in consideration that the tumor might be malignant. The surgical view showed a palpable, elastic, hard mass in the jejunum, necessitating partial resection of the jejunum with 10-cm margins from the tumor on both sides and mesenteric lymphadenectomy. During the lymph node dissection of the small intestinal mesentery, we removed seven lymph nodes, none of which showed signs of metastasis.

**Fig. 3:**
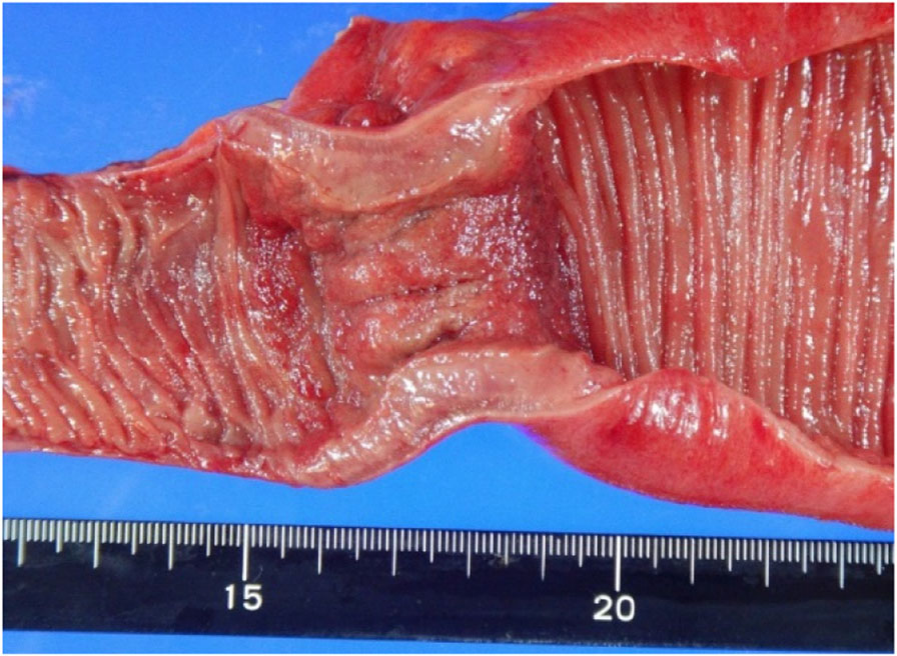
Resected mass measured approximately 60 × 50 mm. The mass, which was elastic and hard, blocked the entire circumference of the elevated cut surface

**Fig. 4:**
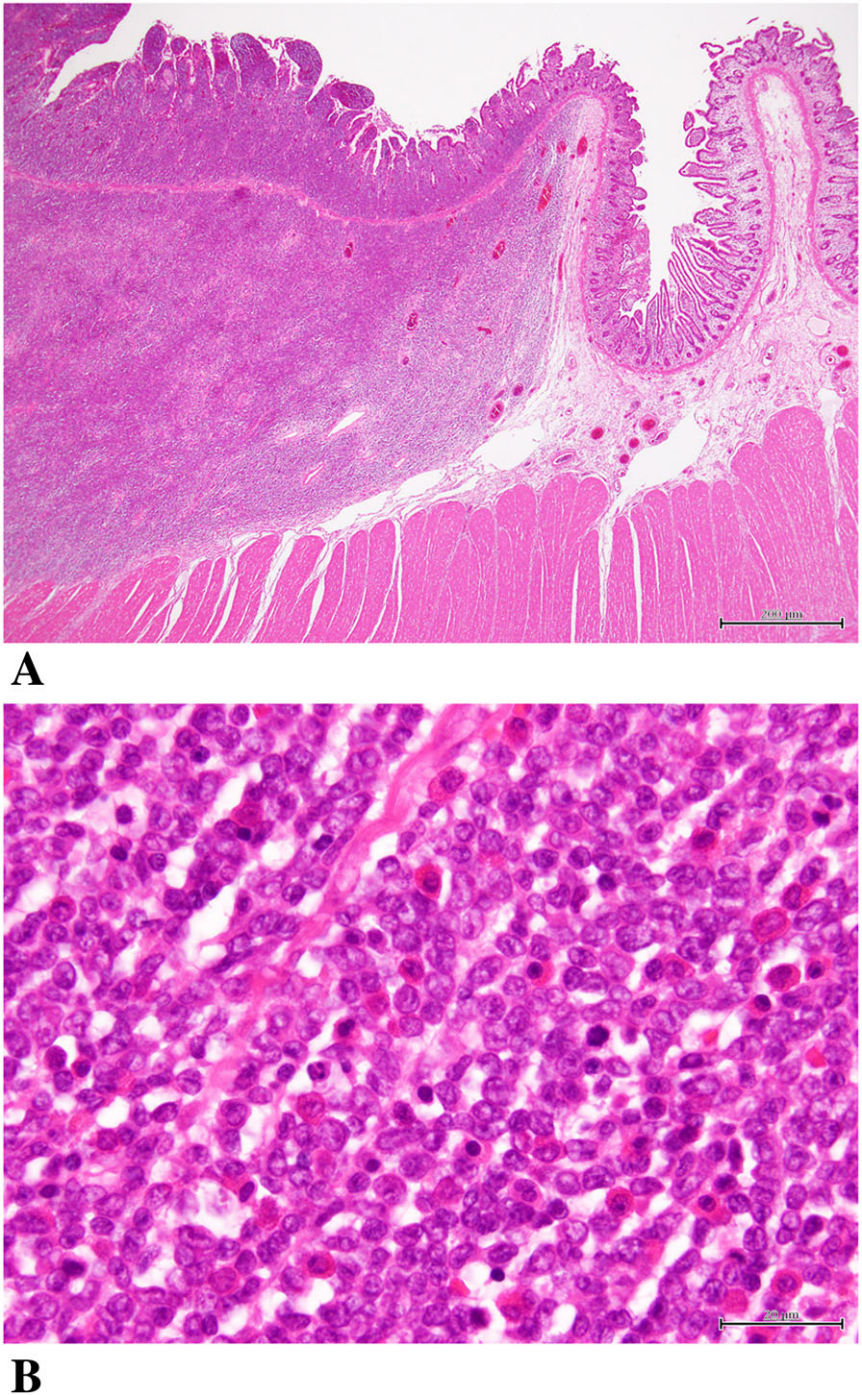
Small intestine specimen shows diffuse infiltration and expansion of immature, atypical cells without tissue destruction (HE; **a** × 20, **b** × 200)

**Fig. 5:**
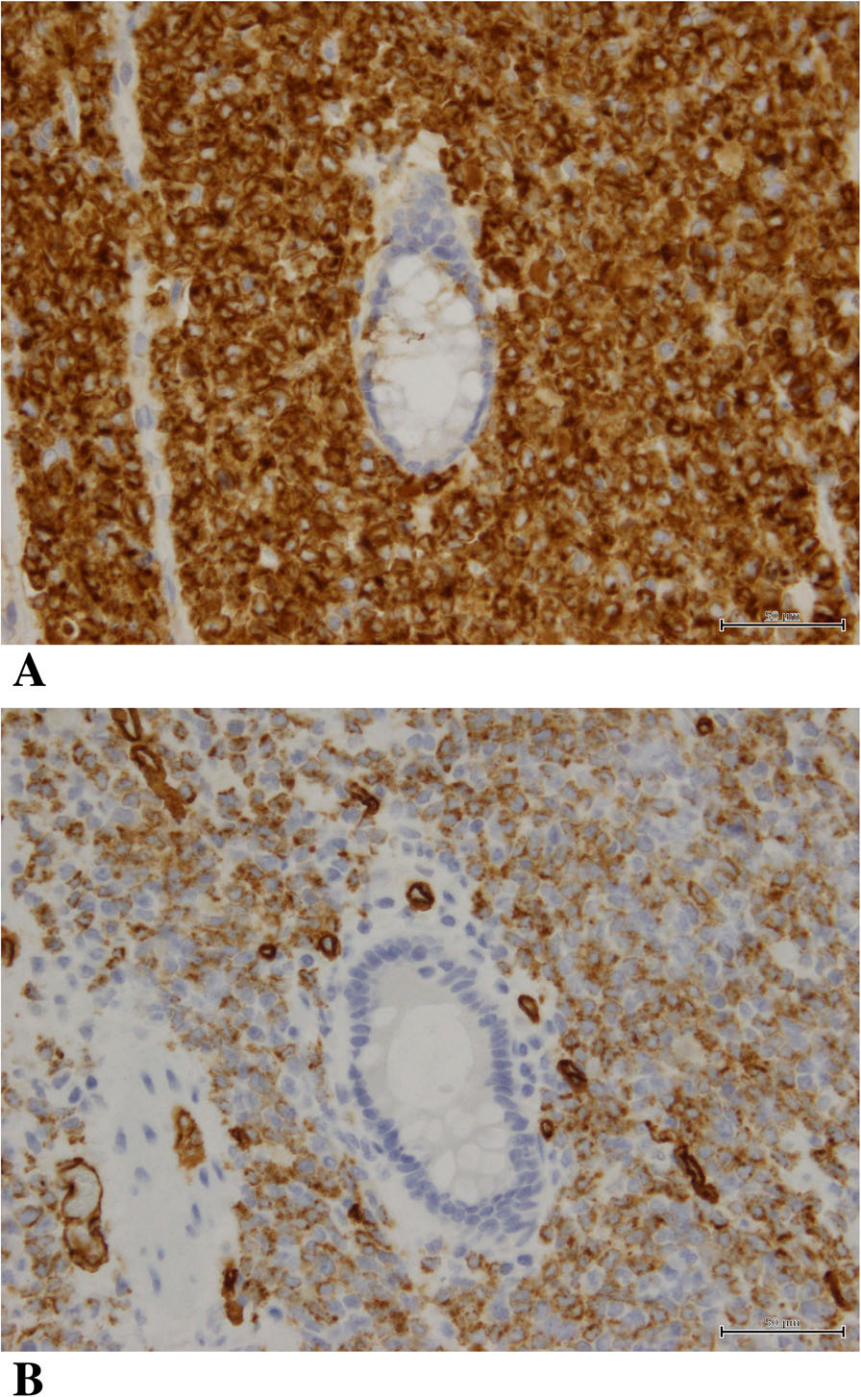
Immunohistochemistry shows staining of the tumor cells for myeloperoxidase (MPO) (**a**) and CD34 (**b**)

Macroscopic examination of the resected specimen revealed a tumor approximately 60 mm in diameter that had caused stenosis of the entire jejunal circumference (Fig. 3). Histologically, hematoxylin and eosin (HE) staining showed diffuse infiltration and expansion of immature, atypical cells but without tissue destruction (Fig. 4). Immunochemical staining revealed that the cells were positive for myeloperoxidase (MPO) and CD34 and negative for CD20 (L26), CD3, CD30 (Ki1), CK (AE1/AE3), CK (MNF116), desmin, CD56, HMB-45, and S-100-protein (Fig. 5). These results confirmed the diagnosis of isolated jejunal MS. The patient’s postoperative recovery was uneventful, and he was discharged from the hospital 9 days after the surgery.

Although the bone marrow aspiration evaluation was negative for cytological findings of leukemia, and ^18^F-fluorodeoxyglucose-positron emission tomography (FDG-PET) showed no abnormal uptake, the patient was started on systemic chemotherapy for AML 6 months after his surgery.

## Discussion

MS occurs as an initial or replacement presentation of AML and sometimes as a complication of myeloproliferative disorders. Isolated MS is rare, with only 1.4–9.0% of AML patients reported to develop MS [3]. Kitagawa et al. reported that the interval between the diagnosis of MS and the occurrence of AML varied from 0.5 to 24 months in patients with initial MS [2]. The male/female ratio is 1.2:1, and the median age was 56 years (range 1 month to 89 years) [4].

Imaging and biopsy results are useful for confirming the diagnosis. FDG-PET shows high uptake by the tumor, and pathological examination shows both diffuse and concentrated areas of hyperplasia with large oval cells [1, 5]. It has been reported, however, that almost half the patients with MS have been misdiagnosed as having a primary or metastatic malignant tumor, especially malignant lymphoma [2, 6]. We did not list MS in the differential diagnosis of the patient and thus arrived at a misdiagnosis. Immunohistochemical examinations (e.g., CD68/KP1, CD33, CD34, CD117, and MPO) are performed to characterize the MS [4]. In our case, MPO was useful for establishing an accurate diagnosis.

Surgical resection is performed to treat an intestinal obstruction, as in our case, but MS patients should be treated with systemic chemotherapy tailored to the AML. Yamauchi and Yasuda analyzed 74 clinical records and showed that the median interval from MS to acute non-lymphocytic leukemia was significantly shorter among the patients who underwent surgical resection with or without irradiation than in those treated with chemotherapy only [6].

MS is rarely seen in the small intestine and certainly would not be easily recognized by general surgeons. Moreover, there is some confusion about the terminology. MS is described as a granulocytic sarcoma (extramedullary myelogenous leukemia) in *Diagnostic Surgical Pathology, 3rd Edition* published in 1999 and as a granulocytic sarcoma or a chloroma in *Surgical Pathology, 10th Edition* published in 2011 [7, 8]. Furthermore, it is called a myeloid sarcoma, granulocytic sarcoma, and chloroma in *Surgical Pathology, 11th Edition* published in 2018 [9]. MS is not even listed In *WHO Classification of Tumours of the Digestive System, 4th Edition* [10]. Hence, it appears desirable that, in the future, the definition of MS be unified regarding terminology.

In most cases of intestinal MS, patients exhibit symptoms of bowel obstruction. A review of the English-language literature between 2002 and 2019 revealed 18 cases of nonleukemic MS of the small intestine (Table 1). Although the type of treatment was not described in 2 cases, the remaining 16 patients underwent surgical resection. Among these 18 patients, 12 were treated with chemotherapy for AML, all of whom experienced complete remission. Among the remaining 6 patients, 4 were treated with surgery only, and 2 of the 4 developed AML. It is important for patients with MS to have an early, accurate diagnosis and to start treatment for AML as soon as possible.

**Table 1:** Nonleukemic MS in the small intestine: summary of studies in the English-language literature

Study	Age/sex	Chief complaint	No. of tumors	First diagnosis	Treatment modality	Outcome
Wang et al. [11]	25/M	Abdominal distension	4	MS	Surgery	Developed AML after 3 months
Cicilet et al. [12]	45/F	Abdominal pain and vomiting	1	MS	Not described	Not described
Hotta and Kunieda [13]	56/M	Vomiting	1	GS	Surgery and chemotherapy for AML	54 months, alive without recurrence
McKenna et al. [14]	49/F	Abdominal pain	1	MS	Surgery and chemotherapy for AML	2 years, alive without recurrence
Palanivelu et al. [15]	52/M	Abdominal distension and pain	1	GS	Surgery	14 months, alive without recurrence
Kumar et al. [16]	55/F	Abdominal pain and vomiting	1	GS	Surgery and chemotherapy for AML	Not described
Yoldaş et al. [17]	44/M	Abdominal pain, distension, nausea, and vomiting	1	MS	Surgery and chemotherapy	9 postoperative months, alive without recurrence
Kwan et al. [18]	39/F	Nausea, vomiting, diarrhea, abdominal pain	1	Crohn's disease	Surgery, steroid therapy, and chemotherapy for AML	2 years, alive without recurrence
Wong et al. [19]	36/M	Abdominal pain	1	GS	Surgery and chemotherapy for AML	1 postoperative year, alive without recurrence
Ioannidis et al. [20]	48/M	Epigastric pain, distension, vomiting	1	MS	Surgery and AML chemotherapy	6 months, alive without recurrence
Lim et al. [21]	55/M	Abdominal fullness and dyspepsia	2	MS	Not described	Not described
Lee et al. [22]	45/M	Abdominal pain	8	GS	Surgery and chemotherapy for AML	12 months, alive without recurrence
Kitagawa et al. [2]	33/F	Abdominal pain and vomiting	2	GS	Surgery and chemotherapy for AML and BMT	57 months, alive without recurrence
Mrad et al. [23]	13/F	Abdominal mass	2	MS	Surgery and chemotherapy for AML	27 months, alive without recurrence
McCusker et al. [24]	22/F	Abdominal pain	2	Large-cell lymphoma	Surgery, CHOP therapy, and chemotherapy for AML and BMT	13 months, alive without recurrence
Kim et al. [25]	49/M	Abdominal pain	5	MS	Surgery	Died
Gajendra et al. [26]	35/M	Abdominal pain	3	T-cell lymphoma	Surgery	Developing AML after 1 month
Jung et al. [27]	48/M	Abdominal discomfort	Not countable	GS	Surgery, chemotherapy for AML and BMT	6 Months, alive without recurrence

*AML* acute myeloid leukemia, *BMT* bone marrow transplantation, *CHOP* cyclophosphamide/hydroxydaunomycin/Oncovin/prednisone, *GS* granulomatous sarcoma, *MS* myeloid sarcoma

## Conclusion

We report the case of an isolated MS that presented with small bowel obstruction. MS is not well known, and many patients with the disease have been assigned an incorrect diagnosis at their first evaluation. Although it is difficult to confirm the diagnosis—especially when MS precedes the occurrence of AML—it is imperative for MS patients to have an early, definitive diagnosis and to start treatment for AML as rapidly as possible. Therefore, we must cite MS as one of the differential diagnoses of small bowel tumors, even in patients without any symptoms of leukemia.

## Abbreviations

MS: Myeloid sarcoma
AML: Acute myeloid leukemia
CT: Computed tomography
HE: Hematoxylin-eosin
MPO: Myeloperoxidase
FDG-PET: ^18^F-fluorodeoxyglucose-positron emission tomography
